# Effect of Inflammatory Cytokines/Chemokines on Pulmonary Tuberculosis Culture Conversion and Disease Severity in HIV-Infected and -Uninfected Individuals From South Africa

**DOI:** 10.3389/fimmu.2021.641065

**Published:** 2021-04-01

**Authors:** Santhuri Rambaran, Kogieleum Naidoo, Lara Lewis, Razia Hassan-Moosa, Dhineshree Govender, Natasha Samsunder, Thomas J. Scriba, Nesri Padayatchi, Aida Sivro

**Affiliations:** ^1^ Centre for the AIDS Programme of Research in South Africa (CAPRISA), Durban, South Africa; ^2^ MRC-CAPRISA HIV-TB Pathogenesis and Treatment Research Unit, Doris Duke Medical Research Institute, University of KwaZulu-Natal, Durban, South Africa; ^3^ Department of Pathology, South African Tuberculosis Vaccine Initiative (SATVI), Institute of Infectious Disease and Molecular Medicine and Division of Immunology, University of Cape Town, Cape Town, South Africa; ^4^ Department of Medical Microbiology, University of KwaZulu-Natal, Durban, South Africa

**Keywords:** inflammation, biomarker, lung cavitation, HIV, Tuberculosis

## Abstract

Novel tuberculosis (TB) prevention and control strategies are urgently required. Utilising specimens from the Improving Retreatment Success (NCT02114684) trial we assessed the associations between inflammatory markers, measured during active TB, with treatment response and disease severity in HIV-infected and uninfected individuals. Multiplex immunoassays and ELISA were used to measure plasma expression of 24 cytokines/chemokines. Cytokines were log transformed to adjust for skewness. We conducted a nested, un-matched, case (n= 31) - control (n=101) study with cases defined as those participants who failed to sputum culture convert within 8-weeks of TB treatment initiation. Additionally, we examined the association between the measured cytokines and time to culture conversion and presence of lung cavitation using cox proportional hazards and logistic regression models, respectively. Multivariable analyses adjusted for a wide range of baseline clinical and demographic variables. IP-10 expression during active TB was associated with increased odds of sputum culture conversion by 8-weeks overall (aOR 4.255, 95% CI 1.025 – 17.544, p=0.046)) and among HIV-infected individuals (OR 10.204, 95% CI 1.247 – 83.333, p=0.030). Increased MCP-3 (aHR 1.723, 95% CI 1.040 – 2.855, p=0.035) and IL-6 (aHR 1.409, 95% CI 1.045 – 1.899, p=0.024) expression was associated with a shorter time to culture conversion in the total cohort. Higher plasma expression of IL-6 (aHR 1.783, 95% CI 1.128 – 2.820, p=0.013), IL-1RA (aHR 2.595, 95% CI 1.136 – 5.926, p=0.024), IP-10 (aHR 2.068, 95% CI 1.034 – 4.137, p=0.040) and IL-1α (aHR 2.008, 95% CI 1.053 – 3.831, p=0.035) were significantly associated with shorter time to culture conversion among HIV-infected individuals. Increased IL-6 and IL-1RA expression was significantly associated with the presence of lung cavitation during active TB in the total cohort (OR 2.543, 95% CI 1.254 – 5.160, p=0.010), (OR 4.639, 95% CI 1.203 – 21.031, p=0.047) and in HIV-infected individuals (OR 2.644, 95% CI 1.062 – 6.585, p=0.037), (OR 7.795, 95% CI 1.177 – 51.611, p=0.033) respectively. Our results indicate that inflammatory cytokines/chemokines play an important role in TB disease outcome. Importantly, the observed associations were stronger in multivariable models highlighting the impact of behavioural and clinical variables on the expression of immune markers as well as their potential effects on TB outcome.

## Introduction

Tuberculosis is the leading cause of death from a single infectious agent with an estimated 10 million new infections reported in 2019 (1). Globally, among HIV-uninfected individuals, an estimated 1.2 million TB deaths occurred, and an additional 208, 000 deaths were recorded among HIV-infected individuals in 2019. Africa accounted for 25% of the TB cases in 2019 and South Africa (SA) is one of eight countries accounting for two thirds of the global burden of TB. SA has the largest HIV epidemic in the world with 7.5 million people living with HIV, 200,000 new HIV infections and 72,000 deaths from AIDS-related illnesses in 2019 (UNAIDS Data, 2020). HIV-infected individuals are 15-22 times more likely to develop TB than HIV-uninfected individuals and TB is a leading cause of HIV-related deaths (WHO, 2019). Furthermore, despite suppressive ART, people living with HIV/AIDS (PLWHA) remain at heightened risk of recurrent TB during their lifetime.

Despite use of TB therapy for many decades, clinical decision making and monitoring of TB treatment response is universally dependent on microbiologic assessment of sputum culture conversion at 2 months post TB-treatment start, despite available data showing low predictive value of two-month sputum tests for predicting treatment failure and relapse (2). Moving away from sputum to more sensitive blood-based biomarkers is imperative for efficient monitoring of treatment efficacy, and early detection of treatment failure.

Measurement of plasma biomarkers could represent a cost-effective, real-time method to determine and understand an individual’s immune status and its effect on TB risk and the subsequent response to TB therapy. A number of studies have examined the effect of soluble plasma cytokine/chemokine responses on TB severity and response to treatment and several cytokines/chemokines such as TNFα, IFNγ, IL-1β, and IP-10 have been linked with disease outcome, presentation or severity (3–6). We have previously identified several inflammatory cytokines (including IL-1β, IL-6 and IL-1RA) associated with risk of TB recurrence in ART treated HIV co-infected cohort (7). Further characterisation of soluble biomarkers, especially in the context of TB/HIV co-infection, will provide valuable insight into the immunological pathways affected and provide new tools for TB screening and monitoring of treatment outcome.

Since HIV is known to cause dysregulation of the TB immune response (8, 9), here we aimed to determine if candidate plasma immune markers detected in individuals with recurrent, active TB disease, were associated with early and late culture conversion and disease severity in TB and TB-HIV co-infected individuals.

## Materials and Methods

### Ethics Statement

Study participants were part of the CAPRISA 011 Improving Retreatment Success trial (IMPRESS, Clinicaltrials.gov, NCT02114684), approved by Medicines Control Council of South Africa (MCC Ref:20130510). Written informed consent was obtained from all study participants prior to enrolment. University of KwaZulu-Natal (UKZN) Biomedical Research Ethics Committee (BREC) reviewed and approved the study protocol (BFC029/13). The nested study protocol was reviewed and approved by UKZN BREC (BREC/00000014/2019).

### Study Participants

Study was performed on stored plasma specimens from CAPRISA 011 study participants that were recruited and treated at an urban clinic (CAPRISA eThekwini Research Clinic) adjoining the largest government outpatient TB facility, the Prince Cyril Zulu Communicable Disease Centre (PCZCDC) in KwaZulu-Natal (KZN), South Africa (SA) (10). All enrolled participants were adults ≥ 18 years, with a previous history of TB and the current diagnosis of rifampicin susceptible sputum smear-positive *Mycobacterium tuberculosis* (MTB) by GeneXpert MTB/RIF^®^ technology. Smear microscopy grading was done using a standardized grading scale: smear 1+ (10 to 99 AFB in 100 fields), smear 2+ (1 to 10 AFB per field in at least 50 fields) and smear 3+ (>10 AFB per field in at least 20 fields) (11). Both HIV-infected and uninfected participants were included in the study. Patients received 8 weeks of intensive phase TB treatment with 2 weekly clinical follow-up visits, and 16-weeks of continuous phase TB treatment with monthly clinical follow-up. We conducted a nested, un-matched case-control study. Cases were defined as those participants who failed to culture convert within 8-weeks of treatment initiation, where culture conversion was defined as the first of two negative sputum cultures at two consecutive visits without an intervening culture positive result. Based on the definition and sample availability, 31 cases and 111 controls were selected for the study.

### Sample Processing

Peripheral blood was collected in acid citrate dextrose (ACD) tubes. Plasma was separated by centrifugation (1600rpm for 10’) and cryopreserved at −80°C until use.

### Cytokine/Chemokine Measurement

Cryopreserved plasma samples were thawed and mixed by vortexing before assays were performed. Cytokine/Chemokine levels were measured using the Millipore Milliplex^®^ assays (Map Human Cytokine/Chemokine Panel I and IV) and analysed on a BioPlex-200 system (Bio-Rad). The Human Cytokine/Chemokine Panel I included the following cytokines and chemokines: pro-inflammatory [IL-1α, IL-1β, IL-6, IL-12(p40), IL-12(p70), TNFα, IFNα2], chemokines (IL-8, IP-10, MCP-1, MCP-3, MIP1α, MIP1β), adaptive (IFNγ, IL-4, IL-15, IL-17α), anti-inflammatory (IL-10, IL-1RA), and growth factors (VEGF).

The Human Cytokine/Chemokine Panel IV included the following cytokines: IFNβ and IL-28B/IFNλ3. Soluble CD14 (sCD14) levels were measured using the Human CD14 Quantikine^®^ ELISA Kit and human Lipopolysaccharide-Binding Protein (LBP) plasma levels were measured using the LBP kit (R&D Systems Inc, USA). All assays were performed following manufacturer’s instructions. Samples with values outside the range of the standard curve were assigned the value half the limit of detection in pg/mL, LOD/2.

### Statistical Analyses

Fisher’s Exact, Chi-Square and Mann-Whitney U tests were used to compare baseline characteristics between cases and controls. All biomarkers with more than 60% of samples above the limit of detection were analysed as continuous variables and log-transformed to adjust for skewness [IFNγ, IL-1β, IL-1RA, IL-6, IL-8, IL-10, IL-12(p70), IL-17α, IP-10, MCP-1, MIP-1α, MIP-1β, TNFα, and VEGF-A]; those with more than 40% of samples below detectability were analysed as binary variables [IFNα2, IL-1α, IL-4, IL-12(p40), IL-15, MCP-3, IFNβ and IFNλ/IL-28]. Logistic regression was used to measure the strength of association between plasma cytokine/chemokine expression at baseline and 8-week culture conversion status and discriminatory ability of the model was quantified using the area under the receiver operating curve (AUC). A Cox proportional hazards model was used to determine the association between cytokine/chemokine expression at baseline and time to culture conversion (first of two consecutive negative TB culture results), measured in days. To determine the association of plasma cytokine expression at baseline with disease severity measured by lung cavitation presence, a logistic regression model was used with presence of lung cavitation at baseline as the predictor outcome and AUC was measured. Multivariable analyses adjusted for a wide range of baseline clinical and demographic variables including study arm, age, sex, HIV status, lung cavitation, alcohol use, smoking and BMI. In addition, when analysing HIV-infected individuals CD4 counts, and viral load were adjusted for. Study arm was excluded in the multivariable lung cavitation analysis as this was not relevant for the studied timepoint. To determine the association between the systemic levels of cytokines/chemokines and bacterial burden at baseline, a one-way ANOVA with Tukey’s multiple comparisons test was done on normally distributed cytokines and non-parametric Kruskal Wallis test with Dunn’s multiple comparisons test was done on not normally distributed cytokines. Statistical analyses were performed using IBM SPSS Statistics version 25, SAS version 9.4 and graphs were made using GraphPad Prism (V8.1.2).

## Results

### Participant Characteristics

A total of 31 cases and 101 control samples were included in the final analysis. Ten controls were excluded from the original analysis as they did not meet the control definition (7 were not TB culture positive at baseline; 1 patient died before the 2^nd^ TB culture negative result; and 2 had inconsistent TB culture results). There were no significant differences between cases and controls for the following characteristics: study arm, age, sex, HIV status, lung cavitation, CD4 count and viral load (**Table 1**). Body mass index (BMI) was significantly higher in controls [cases: 19.28 (IQR 17.96 – 19.98) vs controls: 20.42 (18.64 – 22.95), (p=0.031)]. There was a trend towards higher alcohol (p=0.067) and cigarette use (p=0.051) in cases.

**Table 1:** Demographic and clinical characteristics of study participants.

Variables	Cases n=31	Controls n=101	p-value
**Study Arm n (%)**			
HRZE -Control	18 (58)	45 (45)	.220
HRZM - Active	13 (42)	56 (55)	
**Age (y), median (IQR)**	34 (28 – 43)	36 (31 – 41)	.502
**Sex, n (%)**			
Male	25 (81)	70 (69)	.259
Female	6 (19)	31 (31)	
**Body mass index (kg/m^2^), median (IQR)**	19.28 (17.96 – 19.98)	20.42 (18.64 – 22.95)	**.031**
**HIV status n (%)**			
Negative	11 (35)	25 (25)	.256
Positive	20 (65)	76 (75)	
**CD4 cell count (cells/mm^3^), median (IQR)**	288 (214 – 410)	248 (127 – 413)	.311
**Viral load (copies/ml), median (IQR)**	3453 (20 – 18289)	5878 (20 – 111087)	.386
ARV status* n (%)			
Yes	10 (50)	31 (41)	.609
No	9 (45)	43 (57)	
**Lung Cavities n (%)**			
None	6 (19.3)	33 (32.7)	.317
One Lung	14 (45.2)	42 (41.6)	
Both Lungs	11 (35.5)	26 (25.7)	
**Days to first negative solid culture, median (IQR)^#^**	84 (82 – 91)	42 (28 -55)	**<.0001**
**Alcohol Use in the past 3 months n (%)**			
Yes	13 (42)	24 (24)	.067
**Smoking in past 3months n (%)**			
Yes	15 (48)	29 (29)	.051
**Smear Grade n (%)**			
1+	6 (19.4)	21 (20.8)	.124
2+	2 (6.4)	22 (21.8)	
3+	23 (74.2)	58 (57.4)	
			

*3 missing ARV status.

^#^Measures for all variables, except days to first negative culture are reported at baseline. Significant p-values (<0.05) are bolded.

### Effect of Plasma Cytokines/Chemokines Expression at Active TB on 8-Week TB Culture Conversion

**Figure 1:**
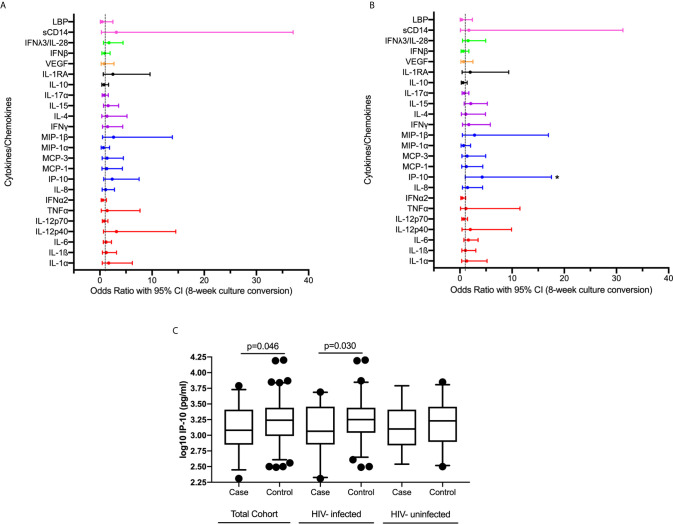
Association between plasma cytokine/chemokine expression at active TB and 8-week culture conversion (n=132) in a **(A)** bivariable and **(B)** multivariable logistic regression model. Individual associations are shown between 8-week culture conversion and pro-inflammatory cytokines (red), chemokines (blue), adaptive response cytokines (purple), anti-inflammatory (black), growth factors (orange), interferons (green) and plasma biomarkers (pink) with error bars depicting 95% confidence intervals. Asterisk indicates significant association (p<0.05). **(C)** IP-10 expression among cases and controls in IMPRESS Cohort (cases n=31, controls n=101) and within the nested group of HIV-infected (cases n=20, controls n=76) and HIV-uninfected (cases n=11, controls n=25) individuals. Cytokines were plotted on log scale (Log 10), Box and Whiskers (5–95%). p-values shown on the graphs are results of the multivariable logistic regression.

We examined the association between plasma cytokine expression during active TB and early culture conversion (8 weeks). We observed no significant association between expression of measured cytokines/chemokines at active TB and TB culture conversion by 8 weeks in bivariable analysis adjusting for study arm (**Supplementary Table 1**, **Figure 1A**). Following multivariable analyses, adjusting for study arm, age, sex, BMI, HIV status, lung cavitation, alcohol use and smoking, we found that increased IP-10 expression was significantly associated with increased odds of early culture conversion [odds ratio (OR) 4.255, 95% CI 1.025 – 17.544, p=0.046] (**Figure 1B**, **Supplementary Table 1**).

We next examined the association between cytokine expression at active TB and 8-week TB culture conversion among HIV-infected individuals while additionally adjusting for CD4 count, and viral load in a multivariable model (**Supplementary Table 2**). An increase in IP-10 expression during active TB was associated with increase in the odds of culture conversion by 8 weeks (OR 10.204, 95% CI 1.247 – 83.333, p=0.030). The levels of IP-10 expression in all individuals and stratified by HIV status are shown in **Figure 1C**. While the sample size was too small to do a detailed analysis for the HIV-uninfected TB patients, IP-10 expression followed the same pattern across all subgroups irrespective of HIV status (**Figure 1C**). These results, in concordance with previously published data highlighting the importance of IP-10 in TB pathogenesis (12–17).

### Effect of Plasma Cytokine/Chemokine Expression at Active TB on Overall Time to Culture Conversion

**Figure 2:**
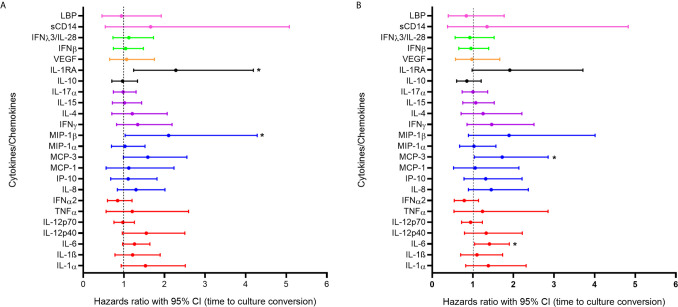
Association between cytokine/chemokine expression measured at active TB and time to culture conversion (n=132) in **(A)** bivariable and **(B)** multivariable models. Individual associations are shown between time to first negative TB culture result and pro-inflammatory cytokines (red), chemokines (blue), adaptive response cytokines (purple), anti-inflammatory (black), growth factors (orange), interferons (green) and plasma biomarkers (pink) with error bars depicting 95% confidence intervals. The dotted line at 1 distinguishes the hazards ratio of higher than 1 (to the right) indicating shorter time to culture conversion and lower than 1 (to the left) indicating longer time to culture conversion. Asterisk indicates significant associations (p < 0.05).

In order to further assess the impact of systemic inflammation during TB active disease on treatment response we used a Cox proportional hazards model to examine the association between cytokine expression during active TB and days to culture conversion (n=132). In the bivariable analysis, increased expression of IL-1RA (p=0.008) and MIP-1β (p=0.041) were significantly associated with shorter time to culture conversion (**Figure 2A**). In the multivariable analysis increased expression of MCP-3 [adjusted hazards ratio (aHR) 1.723, 95% CI 1.040 – 2.855, p=0.035] and of IL-6 (aHR 1.409, 95% CI 1.045 – 1.899, p=0.024) during active TB were significantly associated with shorter time to culture conversion (**Figure 2B**, **Supplementary Figure 1** and **Supplementary Table 3**).

A sub-analysis of HIV-infected individuals was performed adjusting for the effects of viral load and CD4 count (**Supplementary Table 4**). In the bivariable and multivariable models, higher IL-1RA (aHR 2.595, 95% CI1.136 – 5.926, p=0.024) and IL-1α (aHR 2.008, 95% CI 1.053 – 3.831, p=0.035) expression were significantly associated with shorter time to culture conversion. IL-6 (aHR 1.783, 95% CI 1.128 – 2.820, p=0.013) and IP-10 (aHR 2.068, 95% CI 1.034 – 4.137, p=0.040) were significantly associated with shorter time to culture conversion in the multivariable model. Observed increase in inflammatory markers during active TB likely contribute to enhanced cellular responses and faster bacterial clearance.

### Effect of Plasma Cytokines/Chemokines Expression at Active TB on Disease Severity

**Figure 3:**
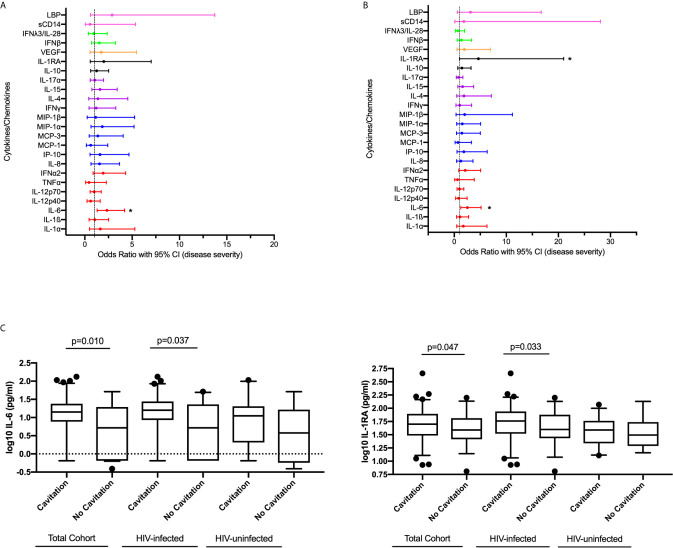
Association between cytokine/chemokine expression measured at active TB and disease severity measured by lung cavitation (n=132) in both **(A)** univariable and **(B)** multivariable model. Individual associations are shown between 8-week culture conversion and pro-inflammatory cytokines (red), chemokines (blue), adaptive response cytokines (purple), anti-inflammatory (black), growth factors (orange), interferons (green) and plasma biomarkers (pink) with error bars depicting 95% confidence intervals. The dotted line at 1 is the interpretation of odds ratio. Asterisk in **(A, B)** indicates significant associations (p < 0.05). **(C)** IL-6 and IL-1RA plasma levels associated with cavitary versus non-cavitary disease in total cohort (Cavitation n=93, no cavitation n=39), HIV-infected participants (Cavitation n=63, no cavitation n=33) and HIV-uninfected individuals (Cavitation n=30, no cavitation n=6). Cytokines were plotted on log scale (Log 10), Box and Whiskers (5–95%). p-values shown on the graph **(C)** are the results of the multivariable logistic regression.

To determine the association between systemic levels of cytokines/chemokines and disease severity measured by the presence of lung cavitation, we compared circulating levels of cytokines/chemokines in all individuals with cavitary and non-cavitary disease using logistic regression. In the bivariable analysis, increased expression of IL-6 (p=0.04) was significantly associated with cavitary disease. IL-6 (OR 2.543, 95% CI 1.254 – 5.160, p=0.010) and IL-1RA (OR 4.639, 95% CI 1.203 – 21.031, p=0.047) positively associated with the odds of lung cavitation in the multivariable model (**Figures 3A, B**, **Supplementary Table 5**).

We performed a sub-analysis of the HIV-infected individuals and adjusted for viral load and CD4 count (**Supplementary Table 6**). Increased expression of IL-6 (OR 2.644, 95% CI 1.062 – 6.585, p=0.037) and IL-1RA (OR 7.795, 95% CI 1.177 – 51.611, p=0.033) were associated with increased odds of cavitation. IL-6 and IL-1RA expression among total cohort and stratified by HIV status in those with and without cavitary disease show similar patterns irrespective of HIV status (**Figure 3C**). These results highlight the dual nature of the host immune response with similar responses being associated with bacterial clearance as well as disease severity and tissue damage.

### Association Between Plasma Cytokine/Chemokine Expression and Bacterial Burden at Active TB

**Figure 4:**
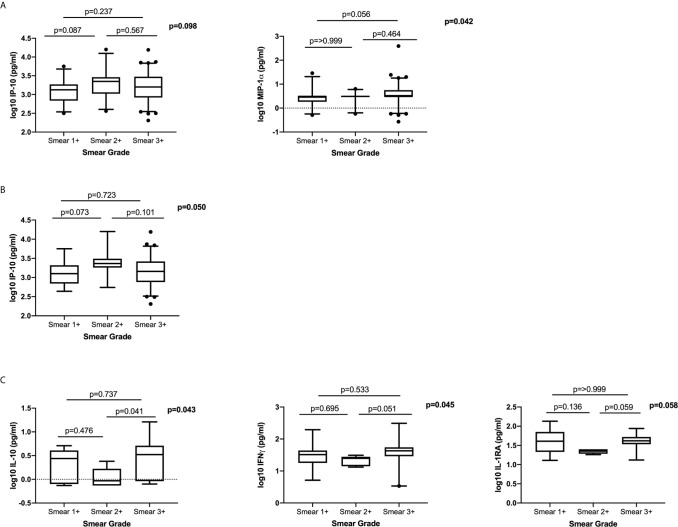
The relationship between plasma cytokines/chemokines and bacterial burden measured by smear grades: **(A)** Total cohort; (Smear 1+ n= 27, Smear 2+ n=24, Smear 3+ n=81), **(B)** HIV-infected (Smear 1+ n=16, Smear 2+ n=18, Smear 3+ n=62) and **(C)** HIV-uninfected (Smear 1+ n=11, Smear 2+ n=6, Smear 3+ n=19). Only cytokines that were significantly different or trending towards significance are shown. Based on distribution IP-10 was analysed using a one-way ANOVA with Tukey’s multiple comparisons test and MIP-1α, IFNγ, IL-10 and IL-1RA were analysed using non-parametric Kruskal Wallis test with Dunn’s multiple comparisons test. Cytokines were plotted on log scale (Log 10), Box and Whiskers (5–95%).

To determine the association between systemic levels of cytokines/chemokines and bacterial burden at active disease, we examined the differences in plasma cytokine/chemokine levels in patients with different smear grades (classified as 1+, 2+ and 3+). We observed no clear dose response with any of the measured cytokines/chemokines and smear grade (**Figure 4**). There was a trend towards higher IP-10 expression in Smear 2+, compared to the Smear 1+ group and trend for higher MIP-1α in Smear 3+ compared to Smear 1+ group (**Figure 4A**). In HIV-infected patients IP-10 levels were higher in Smear 2+ when compared to Smear 1+ group (**Figure 4B**). In the HIV-uninfected group, IL-10, IFNγ and IL-1RA levels tended to be higher in Smear 3+ compared to Smear 2+ group (**Figure 4C**). Our data indicated that measured systemic plasma cytokines/chemokines are not reliable indicators of the bacterial load as measured by the smear grades.

## Discussion

Identification and characterisation of host biomarkers of TB disease severity and treatment response are important tools in progress towards TB elimination and control (18). Here we characterised the role of several plasma cytokines/chemokines, measured during active TB, on early and delayed culture conversion and disease severity in HIV-infected and -uninfected individuals.

Increased IP-10 levels during active TB were associated with early bacterial clearance after 2 months of intensive TB therapy in the total cohort as well as HIV-infected subgroup after adjusting for covariates. IP-10 (also known as CXCL10) is a chemokine that induces chemotaxis, apoptosis, cell growth inhibition and angiostasis (19). A number of published studies have highlighted the role of IP-10 in TB pathogenesis and the potential of using IP-10 as a biomarker of treatment response in TB patients (12–17). Additionally, IP-10 was shown to contribute to the inhibition of mycobacterial replication in the *ex vivo* model of human whole blood assay (20). Due to its stability and high expression, IP-10 has demonstrated potential to be developed into a simple point-of-care (POC) test (21–23). Our data supports these observations and highlights the role of IP-10 in TB clearance among HIV-infected and -uninfected patients with recurrent TB.

After adjusting for covariates, IL-6 and MCP-3 were significantly associated with shorter time to culture conversion in the total cohort. Chemokines, such as MCP-3 play an important role in host response to MTB, with MTB-exposed macrophages showing highly increased MCP-3 expression (24). Furthermore, a strain of BCG that secretes high levels of functional MCP-3 displayed improved immunogenicity and enhanced antigen-specific T cell responses (25). Similar to IP-10, increased MCP-3 levels during active TB likely contribute to enhanced cellular responses and faster bacterial clearance. In addition to its previously identified role as a biomarker of active pulmonary TB (26–28), we found that increased IL-6 expression at active disease is significantly associated with faster bacterial clearance. IL-6, a pleiotropic proinflammatory cytokine, plays an important role in generation of T and B cell responses. Importantly, IL-6 is known to play an important role in protective host immune responses to TB (29, 30) and is essential for generating Th1 cellular responses considered central for MTB control (31). In addition to IL-6 and IP-10, IL-1RA and IL-1α demonstrated an association with shorter time to culture conversion in HIV-infected individuals. IL-1α is an important immunoregulatory cytokine that depending on the magnitude of stress or damage caused by the infection can initiate an inflammatory response or reparative fibrosis (32). IL-1RA is a member of IL-1 family that binds to the IL-1 receptor but does not induce a response (33); its expression is upregulated by inflammatory cytokines including IL-1α and IL-6 as an anti-inflammatory control mechanism (34, 35). IL-1 and IL-1R were shown to be critical for host resistance to MTB (36, 37), while IL-1RA was shown to be a marker of TB disease activity (38, 39). While some of the markers seem to be affected by HIV infection, IL-6 levels are correlated with overall shorter time to culture conversion in both patient groups. Overall, our data shows that the magnitude and nature of inflammatory cytokine expression during active TB disease can be indicative of more efficient cellular response and host’s ability to clear the infection.

Pulmonary cavitation, a hallmark of pulmonary TB, is associated with high bacterial burden and subsequent increase in inflammatory response. Additionally, the host immune response is thought to drive the development of TB cavities (40). Our results indicate that increased plasma IL-6 and IL-1RA levels are associated with cavitary disease in both HIV-infected and uninfected TB patients. Elevated concentrations of both IL-6 (41) and IL-1RA in bronchoalveolar lavage (BAL) fluid were previously found to be associated with tissue necrosis and resulting cavity formation in patients with active pulmonary TB (42). Associations of IL-6 and IL-1RA with bacterial clearance and disease severity (measured by lung cavitation) highlight the dual nature of the host immune response to infections; while immune activation is required for successful pathogen clearance and initiation of protective cellular responses it can also contribute to immune mediated lung pathology and worsened disease outcome.

We observed no clear dose response between, measured plasma cytokines/chemokines and bacterial burden as measured by smear grades. Sputum acid fast bacilli provides an indication of bacillary load and is most often used to monitor TB patients in resource limited settings; however, this method does not distinguish live and dead organisms, has low sensitivity and the specificity predicting treatment failure or relapse is modest (2, 43, 44).

Our study has several limitations, including a relatively small sample size and a clinically complex cohort of patients requiring correction for a wide range of covariates. Future studies should examine cellular phenotypes in order to link the observed inflammatory responses with protective cellular responses to MTB. Our study was focused on drug susceptible TB and future research should also assess immune biomarkers in drug-resistant TB as host immune responses are known to vary between different MTB isolates (45–49). Our study additionally demonstrates that the identified inflammatory markers of disease have low predicative power when considered on their own, with AUC values ranging from 0.56 – 0.65 (**Supplementary Figure 2**). The associations we observed were stronger in the multivariable models highlighting the important effect of other behavioural and clinical variables on the expression of immune markers and their potential confounding effects on TB outcome (50–55).

In summary, our study confirms the importance of inflammatory markers, including IP-10 and IL-6, in TB disease pathogenesis. Further studies are needed to confirm the utility of identified inflammatory markers in TB management. The development and progression of TB disease are influenced by combined effects of various immune as well as behavioural and clinical variables that will have to be considered when utilising immune biomarkers as predictors of risk or protection.

## Data Availability Statement

The original contributions presented in the study are included in the article/**Supplementary Material**. Further inquiries can be directed to the corresponding author.

## Ethics Statement

The studies involving human participants were reviewed and approved by Biomedical Research Ethics Committee, University of KwaZulu-Natal. The patients/participants provided their written informed consent to participate in this study.

## Author Contributions

AS, KN, and SR conceptualized and designed the study. SR and AS performed the experiments. SR, AS, LL, and KN analyzed the data. SN, AS, and KN wrote the manuscript. AS, KN, NS, RH-M, DG, TS, and NP supervised clinical and/or experimental aspects of the study. All authors contributed to the article and approved the submitted version.

## Funding

Research reported in this publication was supported by the Strategic Health Innovation Partnerships (SHIP) Unit of the South African Medical Research Council with funds received from the South African Department of Science and Technology and by the European and Developing Countries Clinical Trials Partnership (EDCTP) (TA.2011.40200.044). SR was supported by the National Research Foundation (Grant Number: 108038). Any opinion, finding, and conclusion or recommendations expressed in this material is that of the author and the NRF does not accept liability in this regard. AS is supported by EDCTP Career Development Fellowship (TMA2016CDF-1582) and NP is supported by EDCTP Senior Fellowship (TMA2018SF-2467).

## Conflict of Interest

The authors declare that the research was conducted in the absence of any commercial or financial relationships that could be construed as a potential conflict of interest.

The handling editor declared a past co-authorship with one of the authors TS.
