# Supplementary material for: Effect of Inflammatory Cytokines/Chemokines on Pulmonary Tuberculosis Culture Conversion and Disease Severity in HIV-Infected and -Uninfected Individuals From South Africa
Source: Front Immunol. 2021 Apr 1;12:641065. doi: 10.3389/fimmu.2021.641065 (PMC8047115; doi:10.3389/fimmu.2021.641065)
Supplement: Supplementary file 1 [file DataSheet_1.docx]

**Supplemental Materials**

**Supplemental Figure 1.** Association between significant plasma cytokine/chemokine at active TB and days to negative culture result (A) Correlation between plasma IL-6 levels and days to negative culture in total cohort, HIV-infected and -uninfected study participants (B) comparison of days to negative culture between individuals with detectable and undetectable MCP3 plasma levels in total cohort, HIV infected and uninfected study participants.

**A.**


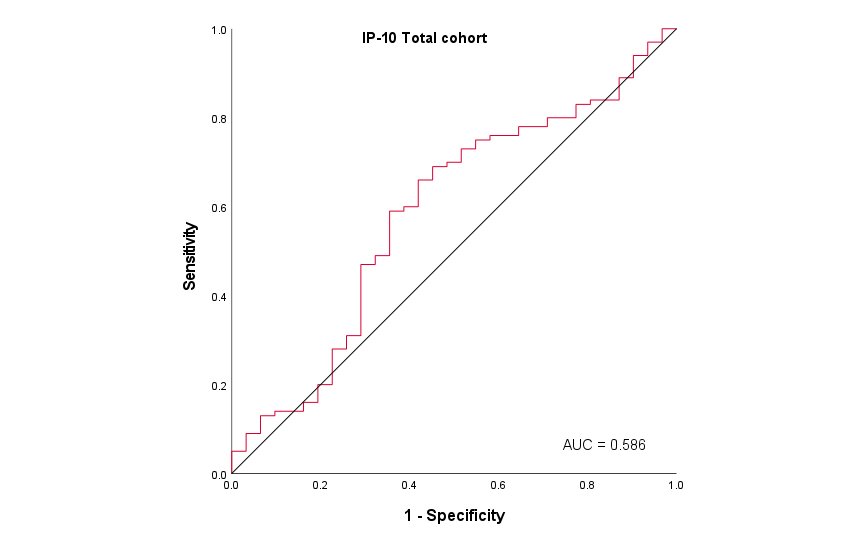

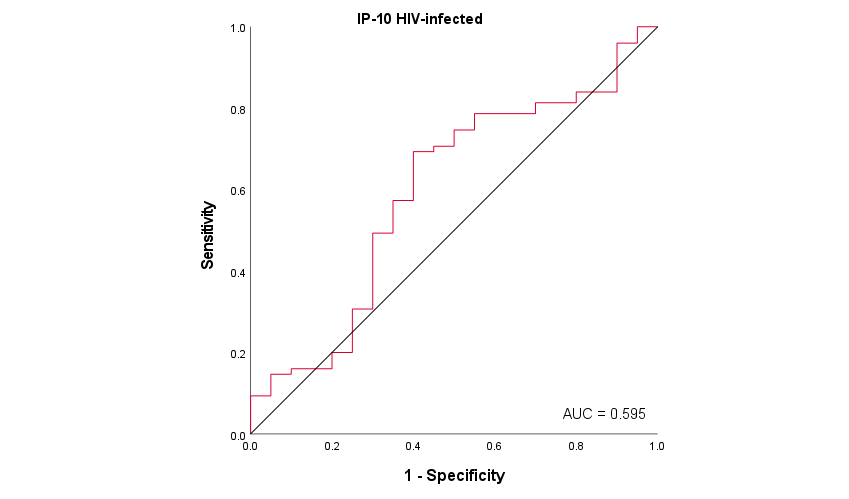


**B.**


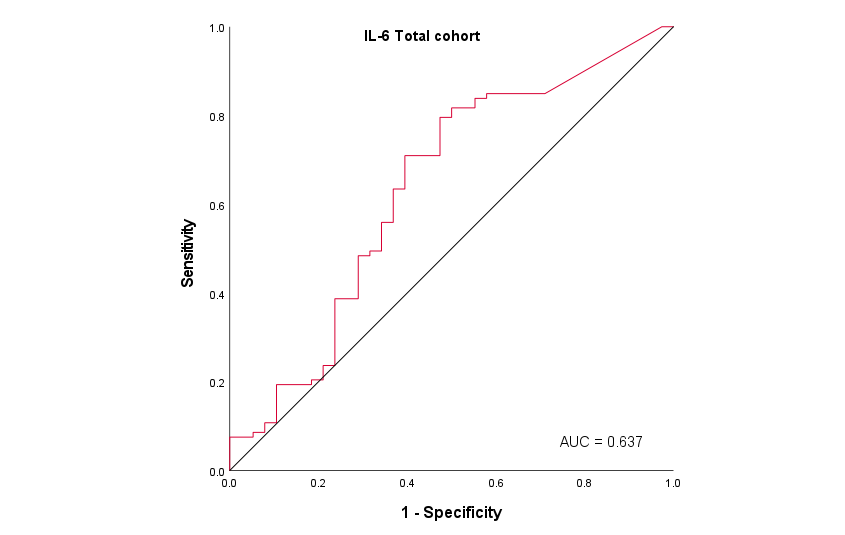

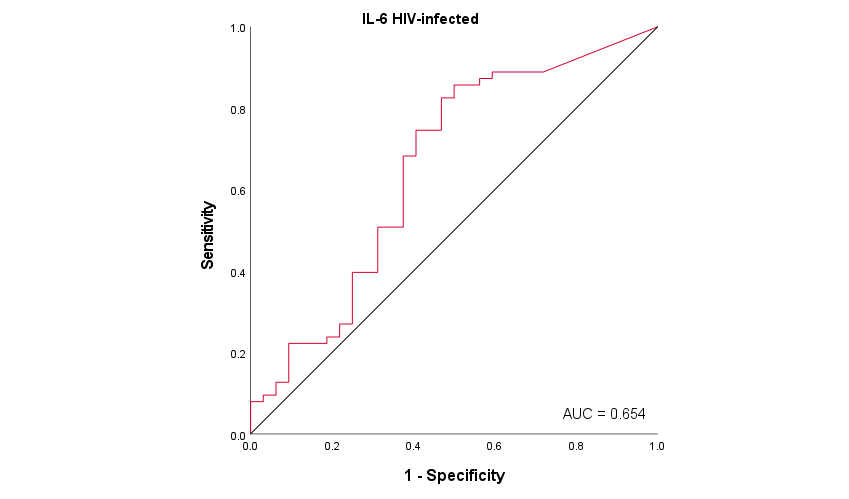


**C.**


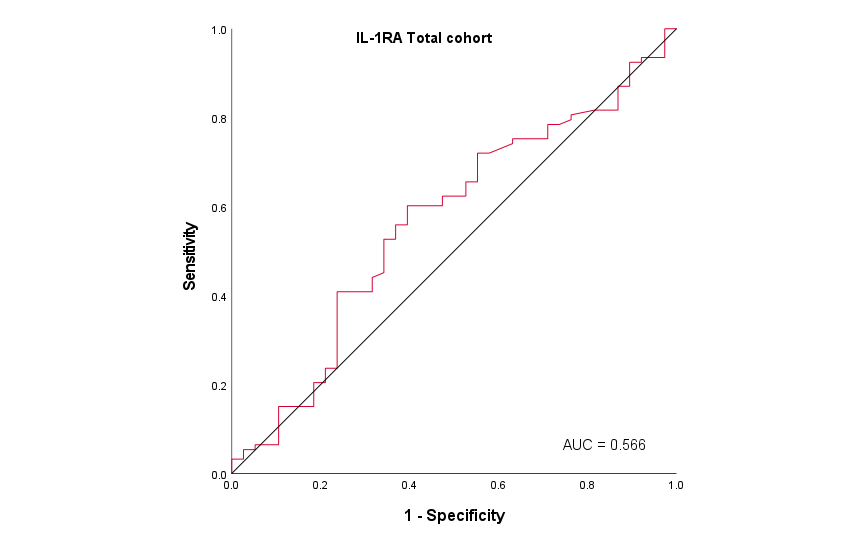

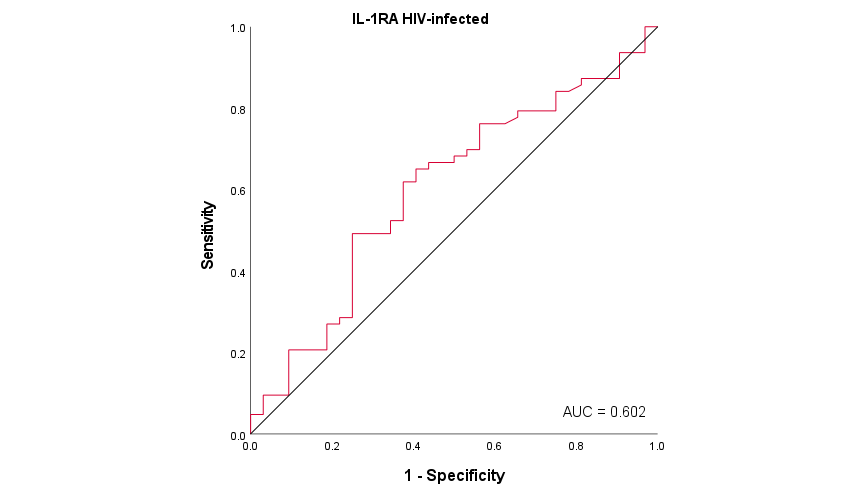


**Supplemental Figure 2.** Receiver-operating characteristic (ROC) analysis. ROC analysis was performed with cytokine/chemokine values as predictors and 8-week culture conversion and disease severity measured by presence of lung cavitation as response variables. (A) chemokine IP-10 as a predictor of 8-week culture conversion, AUC=0.586 (total cohort), AUC=0.595 (HIV infected); (B) IL-6 as a predictor of lung cavitation, AUC=0.637 (total cohort), AUC=0.654 (HIV infected) and (C) IL-1RA as a predictor of lung cavitation, AUC=0.566 (total cohort), AUC=0.602 (HIV infected).

**Supplemental Table 1.** Association between plasma cytokine/chemokine expression at active TB and 8-week culture conversion among total cohort

|  | **Bivariable^b^** | | |  | **Multivariable^c^** | | |
| --- | --- | --- | --- | --- | --- | --- | --- |
| ***Cytokine**  **/chemokine** | **OR** | **CI** | **p-value** |  | **OR** | **CI** | **p-value** |
|  |  |  |  |  |  |  |  |
| IFN-γ | 1.475 | 0.496 – 4.386 | .485 |  | 1.698 | 0.496 – 5.814 | .399 |
| IFN-α2^a^ | 0.555 | 0.244 – 1.262 | .160 |  | 0.422 | 0.164 – 1.087 | **.074** |
| IL-10 | 0.773 | 0.362 – 1.653 | .508 |  | 0.594 | 0.255 – 1.385 | .227 |
| MCP-3^a^ | 1.384 | 0.426 – 4.499 | .589 |  | 1.379 | 0.385 – 4.940 | .622 |
| IL-12p40^a^ | 3.162 | 0.688 – 14.539 | .139 |  | 1.991 | 0.401 – 9.893 | .400 |
| IL-12p70 | 0.850 | 0.470 – 1.538 | .591 |  | 0.718 | 0.355 – 1.453 | .358 |
| IL-15^a^ | 1.577 | 0.695 – 3.580 | .276 |  | 2.070 | 0.818 – 5.238 | .124 |
| IL-17A | 0.831 | 0.428 – 1.613 | .584 |  | 0.847 | 0.419 – 1.709 | .642 |
| IL-1RA | 2.494 | 0.646 – 9.615 | .185 |  | 1.965 | 0.413 – 9.346 | .396 |
| IL-1α^a^ | 1.672 | 0.449 – 6.224 | .443 |  | 1.303 | 0.327 – 5.188 | .707 |
| IL-1β | 1.229 | 0.474 – 3.185 | .672 |  | 1.047 | 0.360 – 3.049 | .932 |
| IL-4^a^ | 1.367 | 0.360 – 5.193 | .647 |  | 1.147 | 0.269 – 4.892 | .852 |
| IL-6 | 1.200 | 0.653 – 2.208 | .556 |  | 1.623 | 0.756 – 3.484 | .214 |
| IL-8 | 1.100 | 0.435 – 2.786 | .840 |  | 1.447 | 0.482 – 4.348 | .509 |
| IP-10 | 2.347 | 0.73 – 7.519 | .152 |  | 4.255 | 1.025 – 17.544 | **.046** |
| MCP-1 | 1.316 | 0.403 – 4.31 | .649 |  | 1.229 | 0.344 – 4.386 | .751 |
| MIP-1α | 0.676 | 0.248 – 1.842 | .444 |  | 0.683 | 0.229 – 2.037 | .494 |
| MIP-1β | 2.618 | 0.491 – 13.889 | .259 |  | 2.809 | 0.465 – 16.949 | .260 |
| TNF-α | 1.397 | 0.253 – 7.692 | .701 |  | 1.149 | 0.116 – 11.494 | .905 |
| VEGF | 0.808 | 0.241 – 2.703 | .729 |  | 0.685 | 0.189 – 2.488 | .566 |
| IFN-β^a^ | 0.892 | 0.379 – 1.962 | .724 |  | 0.624 | 0.227 – 1.718 | .362 |
| IL-28^a^ | 1.752 | 0.692 – 4.436 | .237 |  | 1.565 | 0.496 – 4.941 | .445 |
| sCD14 | 3.155 | 0.271 – 37.037 | .358 |  | 1.718 | 0.094 – 31.25 | .715 |
| LBP | 0.436 | 0.076 – 2.494 | .351 |  | 0.329 | 0.046 – 2.381 | .271 |
|  |  |  |  |  |  |  |  |

Abbreviations: IL, interleukin; IFN, interferon; IP, interferon gamma induced protein; sCD, soluble CD; TNF, tumour necrosis factor; VEFG, vascular endothelial growth factor; LBP, lipopolysaccharide binding protein; OR, odds ratio; CI, confidence interval.

^*^ cytokine values were logged, if not binary

^a^ Analysed as binary variables

^b^ Bivariable analyses adjusted for randomization arm

^c^ Multivariable analyses adjusted for randomization arm, age, gender, HIV status, lung cavitation, alcohol use, smoking and BMI.

**Supplemental Table 2.** Association between cytokine/chemokine expression at active TB and 8-week culture conversion among HIV-infected patients

|  | **Bivariable^b^** | | |  | **Multivariable^c^** | | |
| --- | --- | --- | --- | --- | --- | --- | --- |
| ***Cytokine/**  **chemokine** | **OR** | **CI** | **p-value** |  | **OR** | **CI** | **p-value** |
|  |  |  |  |  |  |  |  |
| IFN-γ | 0.769 | 0.192 – 3.077 | .711 |  | 0.875 | 0.158 – 4.854 | .878 |
| IFN-α2^a^ | 0.645 | 0.234 – 1.783 | .399 |  | 0.601 | 0.181 – 1.997 | .406 |
| IL-10 | 0.835 | 0.348 – 2.004 | .686 |  | 0.519 | 0.181 – 1.490 | .223 |
| MCP-3^a^ | 1.084 | 0.271 – 4.342 | .909 |  | 0.933 | 0.169 – 5.146 | .936 |
| IL-12p40^a^ | 2.372 | 0.492 – 11.440 | .282 |  | 1.182 | 0.196 – 7.136 | .855 |
| IL-12p70 | 0.749 | 0.39 – 1.439 | .386 |  | 0.68 | 0.305 – 1.515 | .346 |
| IL-15^a^ | 1.709 | 0.625 – 4.675 | .296 |  | 2.656 | 0.764 – 9.231 | .124 |
| IL-17A | 0.737 | 0.296 – 1.835 | .512 |  | 0.776 | 0.277 – 2.174 | .631 |
| IL-1RA | 3.030 | 0.595 – 15.385 | .182 |  | 2.179 | 0.299 – 15.873 | .442 |
| IL-1α^a^ | 1.810 | 0.369 – 8.882 | .465 |  | 1.596 | 0.266 – 9.571 | .609 |
| IL-1β | 1.484 | 0.495 – 4.444 | .482 |  | 1.131 | 0.311 – 4.132 | .851 |
| IL-4^a^ | 0.832 | 0.201 – 3.434 | .799 |  | 0.675 | 0.122 – 3.720 | .652 |
| IL-6 | 1.124 | 0.525 – 2.404 | .765 |  | 2.375 | 0.803 – 7.042 | .118 |
| IL-8 | 0.727 | 0.205 – 2.571 | .620 |  | 1.159 | 0.216 – 6.211 | .863 |
| IP-10 | 3.049 | 0.717 – 12.987 | .131 |  | 10.204 | 1.247 – 83.333 | **.030** |
| MCP-1 | 2.994 | 0.445 – 20 | .260 |  | 3.584 | 0.425 – 30.303 | .240 |
| MIP-1α | 0.278 | 0.054 – 1.433 | .126 |  | 0.21 | 0.029 – 1.534 | .124 |
| MIP-1β | 1.096 | 0.145 – 8.333 | .929 |  | 1.399 | 0.128 – 15.385 | .784 |
| TNF-α | 0.786 | 0.089 – 6.944 | .828 |  | 1.017 | 0.056 – 18.519 | .991 |
| VEGF | 0.573 | 0.134 – 2.451 | .453 |  | 0.387 | 0.076 – 1.969 | .253 |
| IFN-β^a^ | 0.782 | 0.286 – 2.136 | .631 |  | 0.477 | 0.127 – 1.792 | .273 |
| IL-28^a^ | 2.401 | 0.854 – 6.752 | .097 |  | 1.577 | 0.445 – 5.589 | .481 |
| sCD14 | 2.681 | 0.092 – 76.923 | .567 |  | 4.484 | 0.114 – 166.667 | .423 |
| LBP | 0.337 | 0.042 – 2.695 | .306 |  | 0.178 | 0.016 – 1.946 | .157 |
|  |  |  |  |  |  |  |  |

Abbreviations: IL, interleukin; IFN, interferon; IP, interferon gamma induced protein; sCD, soluble CD; TNF, tumor necrosis factor; VEFG, vascular endothelial growth factor; LBP, lipopolysaccharide binding protein; OR, odds ratio; CI, confidence interval.

^*^ cytokine values were logged, if not binary

^a^ Analysed as binary variables

^b^ Bivariable analyses adjusted for randomization arm

^c^ Multivariable analyses adjusted for randomization arm, age, gender, CD4 count, viral load, lung cavitation, alcohol use, smoking and BMI.

**Supplemental Table 3.** Association between plasma cytokine/chemokine expression and days to culture conversion

|  | **Bivariable^b^** | | |  | **Multivariable^c^** | | |
| --- | --- | --- | --- | --- | --- | --- | --- |
| ***Cytokine/**  **chemokine** | **HR** | **CI** | **p-value** |  | **HR** | **CI** | **p-value** |
|  |  |  |  |  |  |  |  |
| IFN-γ | 1.341 | 0.822 – 2.189 | .240 |  | 1.466 | 0.858 – 2.506 | .162 |
| IFN-α2^a^ | 0.846 | 0.595 – 1.203 | .351 |  | 0.788 | 0.544 – 1.142 | .208 |
| IL-10 | 0.969 | 0.703 – 1.335 | .846 |  | 0.853 | 0.602 – 1.207 | .369 |
| MCP-3^a^ | 1.590 | 0.990 – 2.553 | **.055** |  | 1.723 | 1.040 – 2.855 | **.035** |
| IL-12p40^a^ | 1.552 | 0.963 – 2.502 | **.071** |  | 1.328 | 0.795 – 2.218 | .279 |
| IL-12p70 | 0.976 | 0.757 – 1.259 | .851 |  | 0.946 | 0.724 – 1.236 | .685 |
| IL-15^a^ | 1.016 | 0.718 – 1.438 | .929 |  | 1.072 | 0.753 – 1.527 | .698 |
| IL-17A | 0.983 | 0.743 – 1.302 | .907 |  | 1.004 | 0.738 – 1.366 | .981 |
| IL-1RA | 2.281 | 1.242 – 4.190 | **.008** |  | 1.910 | 0.982 – 3.713 | **.056** |
| IL-1α^a^ | 1.532 | 0.934 – 2.514 | **.091** |  | 1.383 | 0.826 – 2.314 | .217 |
| IL-1β | 1.217 | 0.782 – 1.894 | .383 |  | 1.102 | 0.701 – 1.733 | .673 |
| IL-4^a^ | 1.206 | 0.703 – 2.069 | .497 |  | 1.254 | 0.712 – 2.210 | .433 |
| IL-6 | 1.261 | 0.968 – 1.642 | **.085** |  | 1.409 | 1.045 – 1.899 | **.024** |
| IL-8 | 1.300 | 0.839 – 2.013 | .240 |  | 1.454 | 0.892 – 2.368 | .133 |
| IP-10 | 1.107 | 0.675 – 1.817 | .687 |  | 1.317 | 0.784 – 2.213 | .298 |
| MCP-1 | 1.121 | 0.562 – 2.235 | .746 |  | 1.060 | 0.526 – 2.135 | .871 |
| MIP-1α | 1.027 | 0.693 – 1.521 | .895 |  | 1.030 | 0.676 – 1.569 | .892 |
| MIP-1β | 2.102 | 1.031 – 4.286 | **.041** |  | 1.892 | 0.892 – 4.013 | **.096** |
| TNF-α | 1.207 | 0.562 – 2.595 | .630 |  | 1.238 | 0.537 – 2.854 | .616 |
| VEGF | 1.068 | 0.651 – 1.752 | .794 |  | 0.977 | 0.574 – 1.664 | .932 |
| IFN-β^a^ | 1.043 | 0.743 – 1.482 | .815 |  | 0.954 | 0.654 – 1.392 | .808 |
| IL-28^a^ | 1.124 | 0.730 – 1.731 | .597 |  | 0.928 | 0.564 – 1.526 | .769 |
| sCD14 | 1.659 | 0.542 – 5.078 | .375 |  | 1.350 | 0.378 – 4.827 | .644 |
| LBP | 0.941 | 0.461 – 1.921 | .868 |  | 0.839 | 0.397 – 1.772 | .646 |
|  |  |  |  |  |  |  |  |

Abbreviations: IL, interleukin; IFN, interferon; IP, interferon gamma induced protein; sCD, soluble CD; TNF, tumour necrosis factor; VEFG, vascular endothelial growth factor; LBP, lipopolysaccharide binding protein; HR, hazards ratio; CI, confidence interval.

^*^ cytokine values were logged, if not binary

^a^ Analysed as binary variables

^b^ Bivariable analyses adjusted for randomization arm

^c^ Multivariable analyses adjusted for randomization arm, age, gender, HIV status, lung cavitation, alcohol use, smoking and BMI.

**Supplemental Table 4.** Association between plasma cytokine/chemokine expression and days to culture conversion among HIV-infected patients

|  | **Bivariable^b^** | | |  | **Multivariable^c^** | | |
| --- | --- | --- | --- | --- | --- | --- | --- |
| ***Cytokine/**  **chemokine** | **HR** | **CI** | **p-value** |  | **HR** | **CI** | **p-value** |
|  |  |  |  |  |  |  |  |
| IFN-γ | 1.225 | 0.667 – 2.248 | .513 |  | 1.704 | 0.831 – 3.498 | .146 |
| IFN-α2^a^ | 0.884 | 0.585 – 1.338 | .561 |  | 0.949 | 0.594 – 1.515 | .876 |
| IL-10 | 0.947 | 0.667 – 1.346 | .763 |  | 0.894 | 0.609 – 1.313 | .568 |
| MCP-3^a^ | 1.546 | 0.885 – 2.700 | .126 |  | 1.620 | 0.847 – 3.097 | .145 |
| IL-12p40^a^ | 1.418 | 0.845 – 2.380 | .186 |  | 1.278 | 0.701 – 2.328 | .423 |
| IL-12p70 | 0.899 | 0.684 – 1.182 | .447 |  | 0.954 | 0.704 – 1.293 | .763 |
| IL-15^a^ | 1.045 | 0.693 – 1.575 | .833 |  | 1.283 | 0.816 – 2.018 | .281 |
| IL-17A | 0.961 | 0.647 – 1.427 | .843 |  | 0.924 | 0.588 – 1.452 | .732 |
| IL-1RA | 2.453 | 1.219 – 4.934 | **.012** |  | 2.595 | 1.136 – 5.926 | **.024** |
| IL-1α^a^ | 1.828 | 1.043 – 3.205 | **.035** |  | 2.008 | 1.053 – 3.831 | **.035** |
| IL-1β | 1.357 | 0.832 – 2.212 | .221 |  | 1.29 | 0.760 – 2.190 | .346 |
| IL-4^a^ | 0.972 | 0.531 – 1.778 | .927 |  | 1.188 | 0.605 – 2.331 | .617 |
| IL-6 | 1.255 | 0.906 – 1.738 | .172 |  | 1.783 | 1.128 – 2.820 | **.013** |
| IL-8 | 1.161 | 0.675 – 1.998 | .590 |  | 1.853 | 0.896 – 3.834 | .096 |
| IP-10 | 1.261 | 0.698 – 2.276 | .442 |  | 2.068 | 1.034 – 4.137 | **.040** |
| MCP-1 | 1.305 | 0.606 – 2.811 | .497 |  | 1.322 | 0.564 – 3.099 | .520 |
| MIP-1α | 0.893 | 0.466 – 1.714 | .735 |  | 0.908 | 0.457 – 1.803 | .783 |
| MIP-1β | 1.768 | 0.760 – 4.113 | .186 |  | 1.926 | 0.739 – 5.019 | .180 |
| TNF-α | 0.950 | 0.379 – 2.381 | .912 |  | 1.54 | 0.619 – 3.828 | .353 |
| VEGF | 1.040 | 0.599 – 1.808 | .888 |  | 0.804 | 0.419 – 1.541 | .511 |
| IFN-β^a^ | 1.097 | 0.726 – 1.658 | .660 |  | 0.911 | 0.565 – 1.471 | .704 |
| IL-28^a^ | 1.229 | 0.775 – 1.947 | .380 |  | 1.015 | 0.585 – 1.760 | .959 |
| sCD14 | 1.957 | 0.472 – 8.118 | .355 |  | 2.553 | 0.534 – 12.217 | .241 |
| LBP | 0.914 | 0.401 – 2.081 | .830 |  | 1.013 | 0.419 – 2.449 | .978 |
|  |  |  |  |  |  |  |  |

Abbreviations: IL, interleukin; IFN, interferon; IP, interferon gamma induced protein; sCD, soluble CD; TNF, tumor necrosis factor; VEFG, vascular endothelial growth factor; LBP, lipopolysaccharide binding protein; HR, hazards ratio; CI, confidence interval.

^*^ cytokine values were logged, if not binary

^a^ Analysed as binary variables

^b^ Bivariable analyses adjusted for randomization arm

^c^ Multivariable analyses adjusted for randomization arm, age, gender, CD4 count, viral load, lung cavitation, alcohol use, smoking and BMI.

**Supplemental Table 5.** Association between plasma cytokine/chemokine expression at active TB and disease severity

|  | **Univariable** | | |  | **Multivariable^b^** | | |
| --- | --- | --- | --- | --- | --- | --- | --- |
| ***Cytokine/**  **chemokine** | **OR** | **CI** | **p-value** |  | **OR** | **CI** | **p-value** |
|  |  |  |  |  |  |  |  |
| IFN-γ | 1.208 | 0.446 – 3.273 | .710 |  | 1.061 | 0.337 – 3.338 | .919 |
| IFN-α2^a^ | 1.946 | 0.878 – 4.313 | .101 |  | 2.098 | 0.868 – 5.068 | .100 |
| IL-10 | 1.248 | 0.618 – 2.519 | .536 |  | 1.477 | 0.674 – 3.236 | .330 |
| MCP-3^a^ | 1.371 | 0.464 – 4.054 | .568 |  | 1.469 | 0.430 – 5.011 | .539 |
| IL-12p40^a^ | 0.609 | 0.230 – 1.616 | .320 |  | 0.829 | 0.278 – 2.469 | .736 |
| IL-12p70 | 0.986 | 0.562 – 1.731 | .962 |  | 1.007 | 0.549 – 1.846 | .983 |
| IL-15^a^ | 1.599 | 0.746 – 3.426 | .228 |  | 1.600 | 0.689 – 3.716 | .274 |
| IL-17A | 1.059 | 0.568 – 1.975 | .857 |  | 0.805 | 0.391 – 1.654 | .554 |
| IL-1RA | 2.005 | 0.573 – 7.016 | .276 |  | 4.639 | 1.023 – 21.031 | **.047** |
| IL-1α^a^ | 1.634 | 0.505 – 5.286 | .412 |  | 1.744 | 0.483 – 6.301 | .396 |
| IL-1β | 1.059 | 0.448 – 2.506 | .896 |  | 1.115 | 0.452 – 2.749 | .813 |
| IL-4^a^ | 1.381 | 0.420 – 4.541 | .595 |  | 1.874 | 0.493 – 7.122 | .357 |
| IL-6 | 2.343 | 1.305 – 4.207 | **.004** |  | 2.543 | 1.254 – 5.160 | **.010** |
| IL-8 | 1.549 | 0.654 – 3.671 | .320 |  | 1.277 | 0.454 – 3.595 | .643 |
| IP-10 | 1.604 | 0.551 – 4.670 | .386 |  | 1.838 | 0.534 – 6.321 | .334 |
| MCP-1 | 0.624 | 0.160 – 2.427 | .496 |  | 0.724 | 0.158 – 3.314 | .678 |
| MIP-1α | 1.847 | 0.656 – 5.202 | .245 |  | 1.548 | 0.474 – 5.054 | .469 |
| MIP-1β | 1.143 | 0.248 – 5.265 | .863 |  | 1.997 | 0.357 – 11.167 | .431 |
| TNF-α | 0.446 | 0.088 – 2.270 | .331 |  | 0.627 | 0.102 – 3.842 | .614 |
| VEGF | 1.735 | 0.551 – 5.458 | .346 |  | 1.936 | 0.540 – 6.943 | .311 |
| IFN-β^a^ | 1.524 | 0.718 – 3.231 | .272 |  | 1.377 | 0.572 – 3.316 | .476 |
| IL-28^a^ | 0.942 | 0.375 – 2.367 | .899 |  | 0.674 | 0.229 – 1.981 | .473 |
| sCD14 | 0.554 | 0.057 – 5.350 | .610 |  | 1.839 | 0.120 – 28.084 | .661 |
| LBP | 2.880 | 0.604 – 13.731 | .184 |  | 3.122 | 0.584 – 16.704 | .183 |
|  |  |  |  |  |  |  |  |

Abbreviations: IL, interleukin; IFN, interferon; IP, interferon gamma induced protein; sCD, soluble CD; TNF, tumour necrosis factor; VEFG, vascular endothelial growth factor; LBP, lipopolysaccharide binding protein; OR, odds ratio; CI, confidence interval.

^*^ cytokine values were logged, if not binary

^a^ Analysed as binary variables

^b^ Multivariable analyses adjusted for age, gender, HIV status, alcohol use, smoking and BMI.

**Supplemental Table 6.** Association between cytokine/chemokine expression at active TB and disease severity among HIV-infected patients

|  | **Univariable** | | |  | **Multivariable^b^** | | |
| --- | --- | --- | --- | --- | --- | --- | --- |
| ***Cytokine/**  **chemokine** | **OR** | **CI** | **p-value** |  | **OR** | **CI** | **p-value** |
|  |  |  |  |  |  |  |  |
| IFN-γ | 1.402 | 0.435 – 4.517 | .571 |  | 0.78 | 0.183 – 3.324 | .737 |
| IFN-α2^a^ | 2.271 | 0.927 – 5.563 | **.073** |  | 2.249 | 0.808 – 6.258 | .120 |
| IL-10 | 1.450 | 0.688 – 3.057 | .329 |  | 1.727 | 0.716 – 4.165 | .224 |
| MCP-3^a^ | 1.481 | 0.431 – 5.082 | .533 |  | 1.828 | 0.439 – 7.609 | .488 |
| IL-12p40^a^ | 0.840 | 0.295 – 2.396 | .745 |  | 1.426 | 0.413 – 4.929 | .641 |
| IL-12p70 | 0.983 | 0.552 – 1.750 | .954 |  | 0.983 | 0.497 – 1.947 | .961 |
| IL-15^a^ | 1.954 | 0.826 – 4.626 | .128 |  | 1.826 | 0.666 – 5.008 | .242 |
| IL-17A | 0.886 | 0.411 – 1.911 | .758 |  | 0.602 | 0.236 – 1.538 | .289 |
| IL-1RA | 2.815 | 0.697 – 11.371 | .146 |  | 7.795 | 1.177 – 51.611 | **.033** |
| IL-1α^a^ | 1.481 | 0.431 – 5.082 | .533 |  | 1.654 | 0.422 – 6.480 | .545 |
| IL-1β | 1.168 | 0.480 – 2.841 | .733 |  | 1.376 | 0.527 – 3.591 | .514 |
| IL-4^a^ | 1.321 | 0.380 – 4.595 | .662 |  | 1.587 | 0.391 – 6.442 | .541 |
| IL-6 | 2.780 | 1.378 – 5.608 | **.004** |  | 2.644 | 1.062 – 6.585 | **.037** |
| IL-8 | 1.702 | 0.605 – 4.789 | .313 |  | 0.971 | 0.243 – 3.880 | .966 |
| IP-10 | 1.615 | 0.485 – 5.370 | .435 |  | 1.351 | 0.269 – 6.791 | .715 |
| MCP-1 | 0.782 | 0.143 – 4.270 | .776 |  | 0.638 | 0.092 – 4.430 | .650 |
| MIP-1α | 2.111 | 0.531 – 8.391 | .289 |  | 2.179 | 0.445 – 10.664 | .336 |
| MIP-1β | 2.160 | 0.385 – 12.128 | .382 |  | 2.745 | 0.360 – 20.903 | .330 |
| TNF-α | 1.014 | 0.163 – 6.296 | .988 |  | 0.493 | 0.060 – 4.019 | .509 |
| VEGF | 1.969 | 0.549 – 7.061 | .299 |  | 1.935 | 0.429 – 8.726 | .390 |
| IFN-β^a^ | 1.315 | 0.563 – 3.072 | .528 |  | 1.006 | 0.345 – 2.928 | .813 |
| IL-28^a^ | 0.688 | 0.264 – 1.791 | .444 |  | 0.451 | 0.136 – 1.499 | .233 |
| sCD14 | 2.057 | 0.126 – 33.652 | .613 |  | 1.971 | 0.077 – 50.757 | .682 |
| LBP | 4.861 | 0.847 – 27.895 | **.076** |  | 5.43 | 0.768 – 38.392 | **.090** |
|  |  |  |  |  |  |  |  |

Abbreviations: IL, interleukin; IFN, interferon; IP, interferon gamma induced protein; sCD, soluble CD; TNF, tumor necrosis factor; VEFG, vascular endothelial growth factor; LBP, lipopolysaccharide binding protein; OR, odds ratio; CI, confidence interval.

^*^ cytokine values were logged, if not binary

^a^ Analysed as binary variables

^b^ Multivariable analyses adjusted for age, gender, CD4 count, viral load, alcohol use, smoking and BMI.
